# Contagious scratching: shared feelings but not shared body locations

**DOI:** 10.3389/fnhum.2013.00122

**Published:** 2013-05-07

**Authors:** Jamie Ward, Vera Burckhardt, Henning Holle

**Affiliations:** ^1^School of Psychology, University of SussexBrighton, UK; ^2^Department of Psychology, University of HullHull, UK

Listening to a lecture on “itching—what's behind it?” can induce observable scratching behavior and self-reported itchiness in the audience (Niemeier and Gieler, 2000). In another study, Papoiu et al. (2011) showed 5 min movies of scratching or rest (either with or without an itch-inducing histamine injection) and noted that watching scratching can increase self-reported itchiness and scratching although the effects tended to be small in participants without a pre-existing dermatological condition.

Previous speculations concerning the neural basis of socially contagious itching have centered on the action-based mirror system (e.g., Ikoma et al., 2006). Recently, Holle et al. (2012) attempted to explore this using fMRI. The stimuli consisted of brief (20 s) movies depicting scratching to the arm or upper chest, and the control movies consisted of tapping the same body part (i.e., the control stimuli involve both a motor act and self-directed touch but imply quite different bodily states). The movies were cropped at the neck to avoid facial expression. The movies depicting scratching were effective inducers of self-reported itch. Participants tested outside the scanner were videotaped and the scratch movies tended to induce scratching behavior (participants in the scanner were instructed not to scratch). The movies depicting scratching (minus tapping) activated many of the regions associated with physically induced itch (via histamine administration) including the premotor cortex, inferior frontal lobe, anterior insula, and primary somatosensory cortex. Thus, contagious scratching is by no means limited to motor-related regions of the brain.

In this commentary, we carry out an additional analysis of the gestures of the videotaped participants in Holle et al. (2012) to examine which aspects of the scratching gesture were reproduced. Two independent raters were asked to determine: (A) whether the participants scratched themselves vs. performed some other body-directed action (e.g., touching); (B) to note the bodily location acted upon; and (C) the hand used. The second rater was blind as to the nature of the visual stimulus presented to the participants and a third rater (again blind) was used to adjudicate between disagreements. Figure 1A shows that when participants observed a movie depicting scratching they were more likely to scratch themselves (χ^2^ = 3.81, *P* < 0.05). That is, both the quality of itchiness (self-reported) and the action of scratching (as observed) is vicariously shared—as already noted by Holle et al. (2012). However, our new analysis shows that other features of the event are *not* vicariously shared. Figure 1B) shows the hand used to perform the scratching action in relation to the hand observed to perform the action^1^. It can be seen that participants use their left and right hands equally often to scratch themselves and this is independent of the hand used in the visual stimulus (χ^2^ = 0.14). Similarly, we coded the part of the body that was scratched. Although the visual stimuli depicted scratching only to the arms and chest (and with cropping at the neck), the vast majority of the participants' own scratches were directed toward their face and hair (see Figure 1C). That is, the bodily location of itching/scratching is not vicariously shared but, instead, gravitates toward the head.

**Figure 1:**
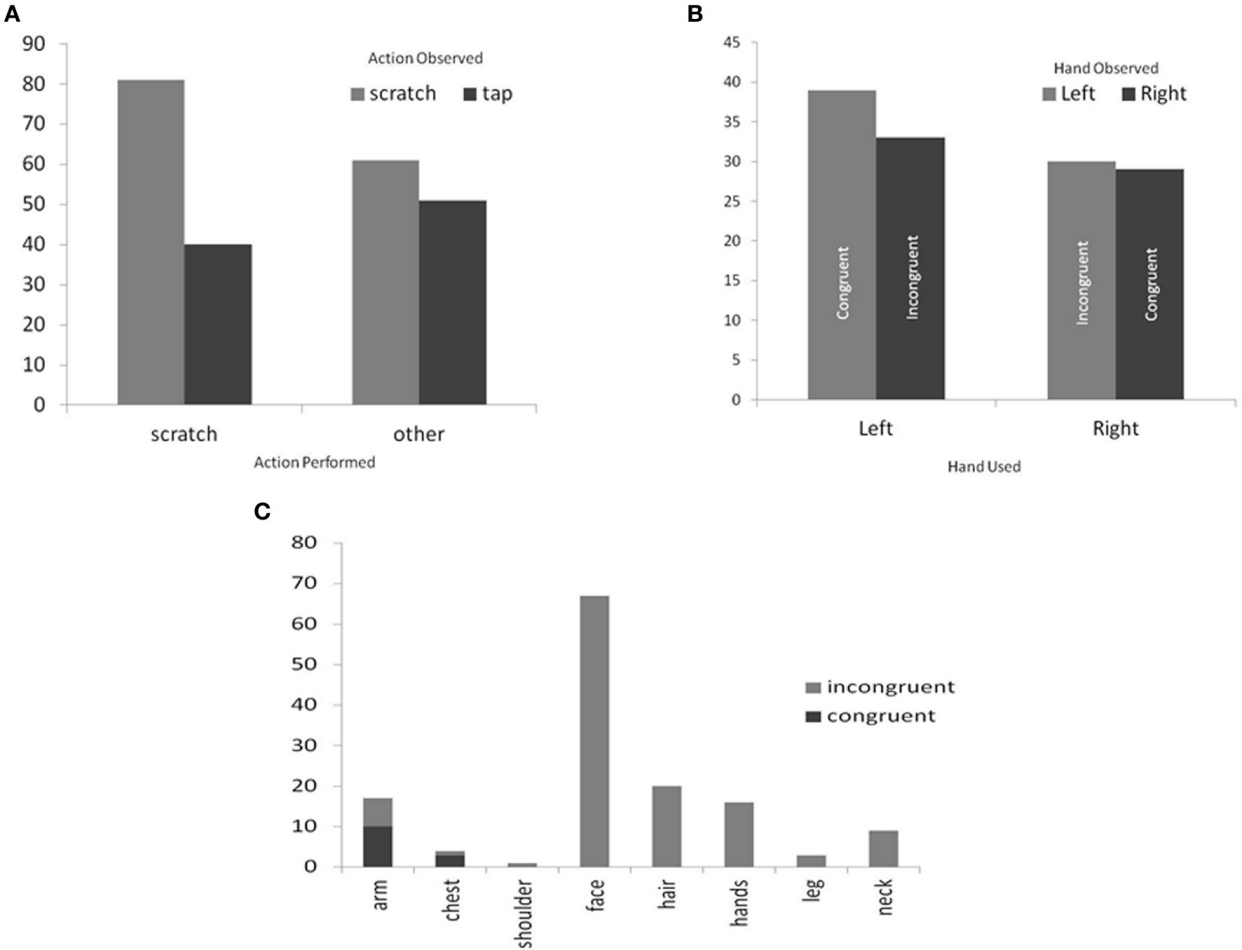
**Frequency counts for (A) different actions performed in relation to the current action observed (B) the hand used to perform scratching in relation to the hand observed and (C) the part of the body that was scratched (note: participants only ever saw the arm and torso scratched)**.

A tendency to scratch body parts distant to that observed was also reported by Papoiu et al. (2011). In that study the participant had been injected with histamine (or saline) in one arm and this would be expected to induce localized itching. In everyday contexts, self-touch (including scratching) is common during social encounters and may be amplified by anxiety (Ekman and Friesen, 1969) or cognitive effort (Barroso et al., 1980) with the hands and face being the most common targets (Goldberg and Rosenthal, 1986). Whatever the reason for the head being the bodily target, our data suggests the driving mechanism behind contagious scratching is related to the processing of affective or sensory quality rather than sharing of bodily locations/effectors. The fact that the anterior insula (involved in affect and interoception) was the only part of the brain to show a sustained response across the duration of the movies depicting itch is consistent with this. Furthermore, non-human primates, who are also susceptible to contagious itch (Nakayama, 2004), show the same pattern of scratching body parts different to the ones observed (Feneran et al., 2013) However, the vicarious perception of itch appears to differ from comparable findings of vicarious experiences of pain (Osborn and Derbyshire, 2010) or touch (Banissy et al., 2009) in response to seeing pain and touch. In both of these cases, there is a direct correspondence between the body observed and the location of vicarious experience (i.e., seeing touch to the arm is felt on the arm), at least in normal-bodied individuals (i.e., non-amputees).

It would be interesting to know whether the bodily target differs between socially induced itch (i.e., vicarious perception) vs. conceptually induced itch (e.g., images of fleas). A more recent behavioral study by Lloyd et al. (2013) used static images of itch-related stimuli (e.g., fleas) and actions (i.e., scratching) and found that these induce both itchiness and scratching relative to neutral control stimuli. Images of bugs on the skin tended to be more potent inducers than images of scratching actions themselves. Again, this is consistent with the idea that contagious itchiness may be more driven by vicarious perception of the feeling state (itchiness/unpleasantness) rather than contagion of the motor act or bodily target.

## Footnotes

^1^Only the actions coded as scratching are displayed, although the pattern is essentially the same when non-scratch actions are analysed.
